# Home-Based Exercise to Improve Motor Functions, Cognitive Functions, and Quality of Life in People with Huntington’s Disease: A Systematic Review and Meta-Analysis

**DOI:** 10.3390/ijerph192214915

**Published:** 2022-11-12

**Authors:** Mohammad Al-Wardat, Tommaso Schirinzi, Hikmat Hadoush, Manal Kassab, Mohammad A. Yabroudi, Józef Opara, Agnieszka Nawrat-Szołtysik, Hanan Khalil, Mohammad Etoom

**Affiliations:** 1 Department of Rehabilitation Sciences, Faculty of Applied Medical Sciences, Jordan University of Science and Technology, Irbid P.O. Box 3030, Jordan; 2 Department of Systems Medicine, University of Roma Tor Vergata, Via Montpellier 1, 00133 Rome, Italy; 3 Department of Maternal and Child Health, Faculty of Nursing, Jordan University of Science and Technology, Irbid P.O. Box 3030, Jordan; 4 Department of Physiotherapy, The Jerzy Kukuczka Academy of Physical Education, 40-065 Katowice, Poland; 5 Department of Physical Therapy and Rehabilitation Sciences, College of Health Sciences, Qatar University, Doha P.O. Box 2713, Qatar; 6 Physical Therapy Department, Aqaba University of Technology, Aqaba 77110, Jordan

**Keywords:** Huntington’s disease, home-based exercise, physiotherapy, motor function, cognition, quality of life

## Abstract

Exercise in different settings has become a fundamental part of Huntington’s disease (HD) management. The aim of this systematic review and meta-analysis was to investigate the effectiveness of home-based exercises (HBE) in HD. Randomized controlled trials (RCTs) investigating the effect of HBE on motor, cognitive, or health-related quality of life (QoL) outcomes in HD were included. Standardized mean difference (SMD), the 95% confidence interval, and *p*-values were calculated by comparing the outcomes change between HBE and control groups. Seven RCTs met the inclusion criteria. The included RCTs prescribed different types of HBEs, i.e., aerobic strengthening, walking, balance, and fine motor exercises. The HBE protocol length was between 6 and 36 weeks. The meta-analyses showed a significant effect of HBE intervention on motor function measure by Unified Huntington Disease Rating and overall QoL measure by Short Form−36 post-treatment respectively, [SMD = 0.481, *p* = 0.048], [SMD = 0.378, *p* = 0.003]. The pooled analysis did not detect significant changes in cognition, gait characteristics, or functional balance scales. The current study shows the positive effect of HBE in HD, especially on motor function and QoL. No significant adverse events were reported. The current results support the clinical effect of HBE intervention on motor function and QoL in HD patients. However, these results should be taken with caution due to the limited available evidence. Well-designed clinical studies that consider the disease severity and stages are required in the future.

## 1. Introduction

Huntington’s disease (HD) is an autosomal dominant inherited degenerative disease of the basal ganglia that has a detrimental effect on physical, cognitive, and psychological status [1]. Individuals with HD display a gradual worsening in mobility and participation, which directly affects their activities of daily living and quality of life (QoL) [2]. The HD course affects the cognitive and psychological status early, while mobility is impacted negatively by several motor manifestations, such as dystonia, chorea, and bradykinesia [2,3]. HD onset is usually between 20 and 60 years of age, and progresses over an average of 20 years [4]. Although the genetic basis of HD is known, however, the genetic mechanisms are still unknown [5]. Indeed, there are some conflicting hypotheses about what is actually toxic (e.g., RNA or protein), as well as about the potential mechanism of toxicity [5]. Thus, the currently available interventions are still limited to symptomatic treatment [4].

Physiotherapy interventions such as balance exercises, aerobic exercise, gait training, breathing exercises, postural control training, and strength exercise have become a fundamental part of managing HD [3,6]. Moreover, previous systematic reviews and randomized control trials (RCT) have shown that exercise improves motor function (gait speed, step length, and walking capacity), balance ability, fitness, and QoL in persons with neurodegenerative diseases such as HD [3,7,8,9,10,11,12,13,14]. Despite these benefits, questions remain regarding the optimal location, supervision amount, delivery approach, duration, intensity, and type of exercise to reach these benefits [15]. This is not surprising because previous research used a wide range of exercises, with different locations, amount, intensity, and amount of supervision [16,17,18,19]. Previous studies reported different types of exercise as individual center-based or structured home-based exercise, fully supervised sessions at a center, minimally supervised at home, or a combination of both supervised center and home exercise [9,16,17,18,19,20]. Since HD is progressive and people with HD have a near-normal life expectancy [21], prescribing sustainable and effective exercise programs for people with HD is essential.

In this context, Home-Based Exercise (HBE) interventions represent a model of care for people with HD and have the potential to be sustained over a long period with minimal or little resources. In addition, they emerged as successful strategies for rehabilitation and physical exercise maintenance even during the COVID-19 outbreak, being substantially appreciated by patients with movement disorders such as Parkinson’s disease [22]. Moreover, a previous systematic review reported a significant beneficial effect for patients with multiple sclerosis [23]. Indeed, HBE (supervised or non-supervised) training represents promising strategies in the therapeutic scenario of neurological diseases [22,23].

However, the current literature provides controversial results and recommendations regarding the effectiveness of HBE for HD patients. To our knowledge, there are no systematic reviews that have specifically investigated the effectiveness, feasibility, and adherence of HBE for people with HD. Indeed, previous systematic reviews have aimed to investigate the effectiveness of physiotherapy interventions (mixed center-based and home-based) on people with HD. Thus, the aim of this systematic review and meta-analysis is to assess the effectiveness of HBE on motor function, cognition, and health-related quality of life in patients with HB.

## 2. Materials and Methods

This systematic review was conducted according to the Preferred Reporting Items for Systematic Reviews and Meta-Analyses reporting guidelines [24].

We followed the PICOS framework to organize the inclusion criteria. Population (P): Adults with Huntington’s disease; intervention (I): predominantly home-based prescribed exercise involved physical practice of exercises targeting gait and/or balance and/or cognitive and/or quality of life, >2 sessions over >2 weeks prescribed by a physiotherapist or health professional, with no or minimal supervision; comparison (C): control group receiving no intervention, usual care, or a placebo intervention, or center-based exercise, where the center-based exercise is equivalent in terms of dose and type of intervention to that of the home-based prescribed exercise; (O): the primary outcome is related to motor ability, and the secondary outcomes include cognitive and quality of life and disability; study design (S): randomized controlled trials (RCTs) published in English language.

Studies meeting any of the following criteria were excluded: (1) Studies including participants with neurodegenerative disease (Parkinson’s disease or Alzheimer’s disease) other than HD; (2) studies published as conference abstracts, dissertations, or in books; and (3) studies without sufficient data to enable pooling of data.

### 2.1. Data Search and Study Selection

A comprehensive computerized search of the MEDLINE via PubMed, Scopus, and Physiotherapy Evidence Database (PEDro) databases was conducted until August 2022. Search and MeSH terms were Huntington’s disease, Huntington’s chorea, chorea, physiotherapy, exercise, home-based therapy, home-based exercise, rehabilitation, exercise therapy, exercise, stretching, strengthening, daily living, activity, mobility, postural control, posture, falls, function, gait, mobility, balance, quality of life, randomized, and quasi-randomized. In addition, the reference lists of included articles were screened for further candidate publications. The PUBMED database search strategy was as follows: (("Huntington Disease" [Mesh] OR Huntington’s Disease OR Huntingtons Disease AND (Exercise [Mesh] OR Exercises OR Physical Activity OR Home-based Exercise OR Home-setting OR Self-exercise OR Physical Therapy OR Physical Exercise OR Physical Therapy modalities [MeSH])) AND (Randomized clinical trial [Publication type]). Study inclusion was decided independently by two authors (M.A. and M.E.) based on the inclusion criteria. 

### 2.2. Data Extraction

Two authors (M.E. and H.H.) independently extracted the following data: (i) demographic characteristics including sample size, age, and disease stage; (ii) HBE dose and type of exercise, percentage of exercise delivered at home, and adherence (the percentage of sessions undertaken/total prescribed sessions); (iii) outcomes used, timing of measurements (pre, post, and follow up), and result at each time point (mean, standard deviation, and number of participants). Disagreements were resolved through discussion or, if required, adjudication by a third author (T.S.). If the original data was unclear or lacking adequate data, the researchers attempted to contact the corresponding authors to provide missing data. 

### 2.3. Risk of Bias and Quality Assessment

The methodological quality of the included trials was assessed by extracting PEDro Scale scores from the (https://www.pedro.org.au (accessed on 16 September 2022)). The PEDro scale includes 10 items for assessment of trial quality based on whether the trials report the randomization procedure, concealed allocation, blinding of patients, blinding of participants, blinding of assessors, adequate follow-up, intention-to-treat analysis, between-group comparability, between-group statistical comparison, and point estimate and variability.

### 2.4. Data Synthesis and Meta-Analysis

Standardized mean difference (SMD), confidence interval (CI) at 95%, and *p*-value were calculated by comparing the change in the study outcomes between HBE and the usual care using the random-effect model of analysis [25,26]. Heterogeneity in effect size was examined by calculating the I^2^ index [27]. The significance level was set at a P of up to 0.05 for the SMD and heterogeneity. Meta-analyses were carried out using the comprehensive meta-analysis, version 2.2.064 software package (Biostat, Englewood, NJ, USA).

## 3. Results

### 3.1. Study Selection

**Figure 1:**
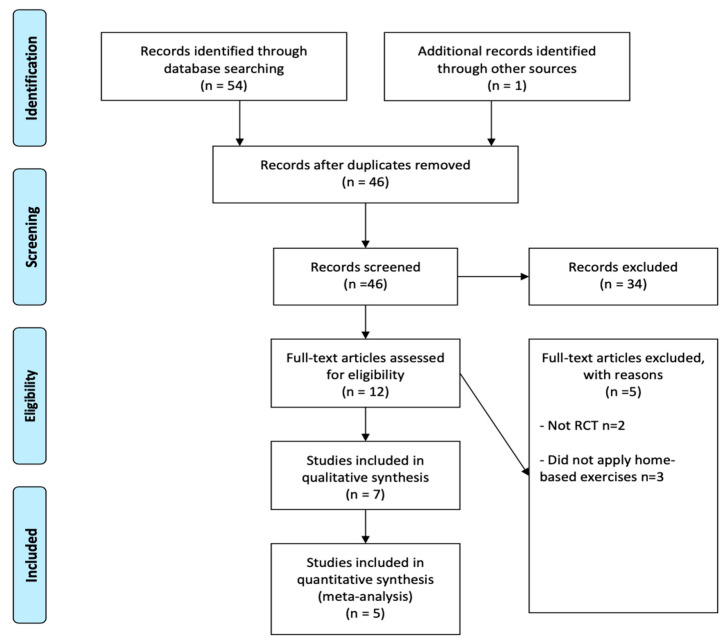
Screening process for systematic review in accordance with PRISMA.

A total of 7 studies were included (Table 1). Databases and hand searches provided 55 publications. After adjusting for duplication, 9 studies have been removed. Based on the title and abstract, 34 articles were excluded; 18 articles were excluded because of the included participants or intervention, and 14 studies because of the study design. Of the remaining 12, 5 articles did not meet the inclusion criteria. Finally, 7 trials met the inclusion criteria, and 5 were included in the meta-analysis (Figure 1).

### 3.2. Study and Participant Characteristics

A total of seven trials (Table 1) constituted 166 participants compared HBE with usual care or placebo. The mean age of the participants across the trials ranged between 50 and 57 years, with a reported standard deviation (SD) between 2.75 and 14.7 years. All the included studies enrolled both male and female participants except one study [9], where the gender of the participants was not specified. Five studies reported disease severity, three studies included participants with early to moderate disease stages [10,28], one study included participants with mild to moderate disease stages [9], and one study included participants with moderate disease stages [18]. 

### 3.3. Quality and Risk of Bias

The mean PEDro score of the studies was 6 (range: 3–7) (Table 1). The outcome assessors were blinded in all articles except two [9,17]. All trials carried out an intention-to-treat analysis except [29], and four studies [10,17,18,19] reported concealed allocation.

### 3.4. Intervention

The included studies prescribed different types of exercises, including multimodal training in five studies (i.e., aerobic (cycle ergometer), resistance (machines) strengthening exercises, walking, balance, and fine motor exercises) [9,10,18,20,28]. Two studies involved video game dance exercises [29] and resistive inspiratory and expiratory muscle training [17]. The intervention protocols used in the included studies lasted between 6 and 36 weeks. Training frequency ranged between 2–6 sessions per week with a duration of 25–75 min per session. Interventions used DVD [9], exercise manual [20], and self- or coach-guide [10,17,18,28,29].

### 3.5. Effect of HBE on Primary Outcomes (Motor Function Ability)

**Figure 2:**
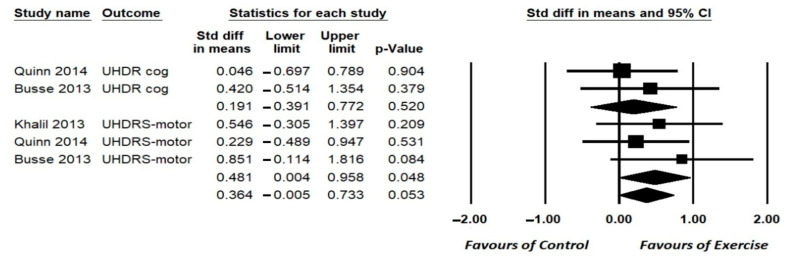
Forest plot for the effects of Home-based exercise (HBE) intervention compared with conventional interventions on Unified Huntington Disease Rating Scale (UHDRS) motor and cognitive sections [9,10,18]. CI, confidence interval.

A total of four studies assessed motor function ability using the Unified Huntington Disease Rating Scale (UHDRS), of which three found a beneficial effect of HBE [9,18,28] and one found no significant changes [10]. Three studies [9,10,18] could be meta-analyzed, including 86 participants, with pooled analysis showing a small significant effect of HBE on motor function [SMD = 0.481, 95% (CI) = −0.004 0.958; *p* = 0.048], I^2^ = 85.2%, *p* = 0.000 (Figure 2).

**Figure 3:**
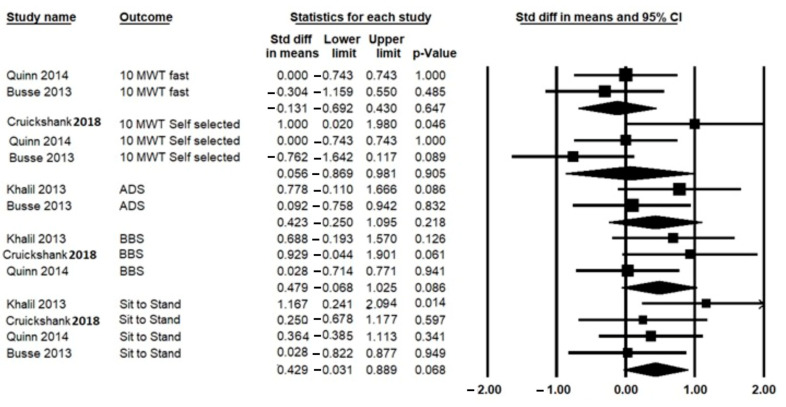
Forest plot for the effects of Home-based exercise (HBE) intervention compared with conventional interventions on gait speed. 10-MWT: 10-m walk test [10,18,20]; ADS: average daily steps [9,10]; BBS: Berg Balance scale [9,18,20] and sit-to-stand test [9,10,18,20]; CI: confidence interval.

A total of five studies measured gait speed, of which three studies used the Timed 10-Meter Walk Test (10-MWT) self-paced and fast-paced [10,18,20], and two studies used the GAITRite system [9,29]. The pooled analysis includes 79 participants showed no significant differences between HBE and the control group after intervention (SMD = 0.056; 95% CI = −0.869 to 0.981; *p* = 0.905), I^2^ = 0%, *p* = 0.261 (Figure 3). 

A total of six studies reported the effect of HBE on the Berg Balance Scale (BBS), Romberg test, and Activities-Specific Balance Confidence Scale [9,10,18,20,28,29]. The pooled SMD, including 73 participants, was not improved significantly after HBE interventions for BBS (SMD = 0.479; 95% CI = −0.068–1.025; *p* = 0.086), I^2^ = 87.6%, *p* = 0.000) (Figure 3).

A total of four studies, including 104 participants, analyzed the effect of HBE on the sit-to-stand test [9,10,18,20]. There was no significant difference between the HBE and the control groups regarding sit to stand test after the intervention (SMD = 0.429; 95% CI = −0.031 to 0.889; *p* = 0.068), I^2^ = 74%, *p* = 0.001) (Figure 3). 

A total of two studies, including 56 participants, reported the effect of HBE on the average of active daily steps (ADS) [9,10], with pooled results indicating no significant effects of HBE on ADS (SMD = 0.423; 95% CI = −0.250 to 1.095; *p* = 0.218), I^2^ = 68%, *p* = 0.003) (Figure 3).

### 3.6. Effect of HBE on Secondary Outcomes

#### 3.6.1. Cognition

A total of three studies assessed different measures of cognition (e.g., Symbol Digit Modalities Test (SDMT), Hopkins Verbal Learning Test-Revised (HVLT-R), Colour Word Interference), with two used UHDR-cognitive scales reporting no beneficial effect of HBE [10,18] and one finding significant changes [28]. A total of two studies, including 61 participants [10,18], could be meta-analyzed, with pooled analysis showing no significant effect of HBE on cognitive abilities [SMD = 0.191, 95% (CI) = −0.391–0.772; *p* = 0.520], I^2^ = 50%, *p* = 0.71 (Figure 2).

#### 3.6.2. Health-Related Quality of Life

**Figure 4:**
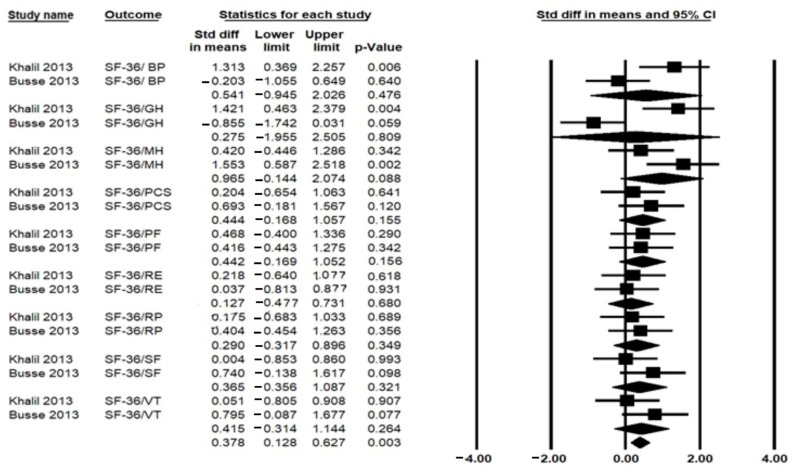
Forest plot for the effects of Home-based exercise (HBE) intervention compared with conventional interventions on quality of life [9,10] measured by SF-36: 36-Item Short Form Health Survey. BP: Bodily Pain; GH: General Health Perception; MCS: Mental Component Summary; MH: Mental Health; PCS: Physical Component Summary; PF: Physical Functioning; RE: Role limited due to emotional problems; RP: Role limited due to physical problems; SF: Social Functioning.

A total of six studies assessed QoL, of which two used the 36-Item Short Form Health Survey (SF-36) [9,10], one used the swallowing quality of life questionnaire [17], one used the EuroQoL 5-item questionnaire and the Huntington’s Disease Health-related quality of life (HDQoL) [18], one used the World Health Organization Quality of Life—Bref (WHOQOL-Bref) assessment [29], and one used the SF-36v2 Health Questionnaire and Huntington’s-Disease Quality of Life Battery for Carers [28]. A total of two studies used SF-36, including 56 participants, which could be meta-analyzed. The pooled analysis revealed significant effects of HBE on overall QoL [SMD = 0.378, 95% (CI) = 0.128–0.627; *p* = 0.003], I^2^ = 27.8%, *p* = 0.239 (Figure 4). However, the pooled analysis of the SF-36 domains (bodily pain, general health perception, mental component summary, mental health, physical component summary, physical functioning, role limitation due to emotional problems, role limitation due to physical problems, and social functioning) showed no significant effect in favor of HBE (Figure 4).

### 3.7. Adherence

All the included studies reported adherence to HBE except one [29]; the weighted average of adherence was 78.48% (range 56–100%).

## 4. Discussion

This review and meta-analysis is the first to systematically evaluate the effectiveness of HBE on motor function, cognition, and QoL among patients with HD. It included seven RCTs that investigated the effectiveness of HBE on motor function, cognition, and QoL in patients with HD compared with control interventions. The pooled analysis results showed significant improvements in favor of HBE in two outcomes: UHDR motor scale and general QoL (SF-36). There were no significant improvements in the UHDR cognitive scale, balance, gait speed, and QoL domains.

UHDRS scores are recommended for assessing the severity of motor signs in HD [30]. As the UHDRS was the primary outcome of this study, three of the included articles and meta-analysis found that the HBE significantly improved UHDRS compared with control interventions. Despite this significant improvement in UHDRS in favor of HBE, these results should be taken with caution due to the small number of included studies and participants. In line with our results, previous clinical studies reported a significant improvement in UHDRS in favor of physiotherapy interventions, including conventional exercise [10,28,31,32]. Despite these data regarding the effectiveness of exercise interventions, the results of the previous systematic reviews and meta-analyses are controversial. For example, Playle R et al. (2019) concluded that there is no evidence of an exercise effect on the UHDRS motor scores [33]. However, Fritz NE (2017) reported a beneficial effect of exercise and physical activity interventions in HD concerning motor function [7]. Our pooled analysis found that HBE significantly improved motor function in patients with HD.

There are several possible explanations for these improvements in the UHDR motor scale in favor of HBE interventions. First, multiple lines of evidence reported that muscle strength is reduced in patients with HD and contributes to impaired motor function [34,35]. It is plausible that HBE interventions have positive and beneficial effects on muscle mass and neural drive. Second, previous studies reported a significant effect of exercise on cardiorespiratory functions in HD [36,37]. In addition, it has been suggested that oxygen uptake during exercise and motor functioning in HD may positively improve HD progression. Third, the average adherence rate to HBE protocols was 78%, and no side effects were reported after HBE. Indeed, HBE programs seem more valuable in terms of cost and patient accessibility. In addition, healthcare providers may consider the location of the HBE program according to the patient’s preference, motivation, and available resources. Therefore, it could be that the enjoyment of HBE is reflected in motor function improvements.

This analysis found significant improvement in overall QoL measured by SF-36. However, the pooled analysis of SF-36 domains revealed no significant improvement in favor of HBE interventions. While there is evidence that physiotherapy interventions improve QoL [38], this has not been confirmed [33]. Previous meta-analysis focused on supervised and self-directed exercise for HD [33] found no significant improvement in QoL. These controversial results might be explained by the poor sensitivity of QoL outcome measures [39]. Moreover, HBE interventions (in terms of duration, frequency, and intensity) might not have been enough to influence QoL in HD. This is not surprising; indeed, there is a lack of precise information on the disease duration and severity of patients to be included in HBE programs. Additionally, the included studies have a high heterogeneity in types of exercises, intensity, frequency, and supervision rate. We postulate that this heterogeneity may influence the results. Future studies are recommended to provide longer duration of HBE interventions and should consider frequency and intensity according to disease severity.

Contrary to our expectations, the pooled analysis did not detect any significant results regarding the other parameters, such as UHDR cognitive, 10-MWT, BBS, ADS, and chair sit-to-stand test. These results are in line with the previous meta-analysis of RCTs indicated no significant effects from exercise on gait, balance, and cognition [33]. It is important to mention that these results may be because of the small number of included studies and the high heterogeneity of the HBE intervention parameters. In addition, we expect that the included studies’ intensity and durations were insufficient to improve balance, other gait characteristics, and cognitive abilities. Moreover, the included studies lack the assessment of long-term follow-up; indeed, just 2 studies among the included studies assessed the long-term effect of HBE interventions [17,18]. Thus, it’s possible that increasing the frequency and duration of HBE interventions and assessing the long-term effect of interventions would lead to improved outcomes. Indeed, the effectiveness of exercises on HD progression is still unclear. Considering the nature of the disease, the role of exercise in stabilizing or slowing down the nature of HD progression needs more investigation. In addition, one of the important factors that may influence the effectiveness of HBE is adherence to exercise. Despite the included studies reporting a 78.48% adherence average, it is still difficult to guarantee full exercise adherence due to many barriers. The nature of HD, commitment of caregivers, forgetfulness, and the boring perception of exercise may affect the adherence rate [40]. Thus, adherence to HBE should be considered according to disease severity and stage, especially for long-term HBE protocol. In addition, we propose that the HBE protocols reviewed here were not adapted to HD severity and stage. Indeed, the current available evidence supports HBE in the early and/or middle stages of HD. Thus, future studies are recommended to select the HBE interventions according to disease severity and disease stage. These results suggest that more clinical research is urgently needed to understand what contexts HBE may induce an optimal effect on the gait, balance, cognition, and QoL in HD.

Different lines of evidence confirmed the impact of exercises on both cellular and molecular mechanisms. For example, physical exercise increases the Brain-Derived Neurotrophic Factor (BDNF)-serotonin axis at the peripheral or central level [41]. BDNF has a significant effect as a neurotrophic and neuroprotective action within the brain [42]. In addition, the BDNF action is obvious within the serotoninergic system; thus, it improves synaptic activities and increases the transmission in the cerebral cortex, basal ganglia, and brainstem [43]. The effect of exercise interventions on brain circulation should be taken into consideration. Indeed, brain blood perfusion improves post-exercise, which may prevent the vascular and cerebral amyloidopathy responsible for brain neurodegenerations [14,44]. Therefore, it’s important to mention that the included studies applied different types of HBEs as aerobic strengthening, walking, balance, and fine motor exercises. We propose that HBEs could exert beneficial effects on neurodegenerative processes in HD. More clinical studies investigating the effect of HBE on both cellular and molecular mechanisms in HD patients are required.

This systematic review has different limitations. The main limitation of most of the included studies were the heterogeneous HBE protocols (intensity, frequency, and duration) and most of the outcome measures. Indeed, this does not allow us to estimate and recommend the ideal HBE protocol for HD. Standardizing the HBE protocol in future studies is highly recommended. One obvious limitation is the small and limited number of the included studies, which prevents us from performing subgroup analysis based on supervision rate and type of exercise. Further studies are needed to clarify if the supervision rate may influence the improvement of HBE (partial supervision vs. full supervision). Another limitation is that the participants (mild to moderate HD) in the included studies do not represent the general population of patients with HD. Therefore, future clinical-powered studies are urgently required to develop appropriate HBE interventions for patients with HD in the later stage of the disease. Finally, HD is a neurodegenerative disease with a long course of progression. Thus, future studies are recommended to assess the effect of HBE intervention in long-term follow-up. Despite these limitations, the quality of the included studies is high.

## 5. Conclusions

In conclusion, the results of this systematic review and meta-analysis support the clinical effect of HBE intervention on motor functions and QoL in HD. However, these results should be taken with caution due to the limited available evidence. We recommend that healthcare providers incorporate HBE intervention into the routine management of patients with HD who do not have specific contraindications. Well-designed clinical studies that consider the disease severity and stages are required in the future.

## Figures and Tables

**Table 1:** The summary and characteristics of the included studies.

Study	PEDro Total Score	Participants, Demographical, and Clinical Data	Outcome Measurements	Type and Duration of Intervention	Main Results	Adherence Rate
Khalil et al. 2013	5	N = 25 Age = 52.5 ± 13.5 Disease stage = Mild to moderate HD	UHDRS-MS; Gait speed using GaitRite, BBS, Chair sit to stand test, average of daily steps and SF-36.	Exp: supervised home exercise program using a DVD and a walking program. One home visit to teach the program and then weekly follow-up phone calls. CG: continued usual care. 75 min × 3 times a week × 8 weeks	Exp group had significant improvements in gait speed, balance sit-to-stand test, average daily steps, and UHDRS-mMS. No significant changes in the SF-36.	80%
Kloos et al. 2013	3	N = 24 Age = 50.7 ± 14.7 Disease stage = NR	ABC scale; WHOQOL-Brief	Exp: participants played the video game Dance Revolution with therapist supervision in homes. CG: played a handheld video game unsupervised. 45 min × 2 times a week × 6 weeks	No significant changes in the ABC scale and WHOQOL Brief.	NR
Reyes et al. 2015	7	N = 18 Age = 56 ± 10.2 Disease stage = NR	Swallowing QOL questionnaire	Exp: home-based resistive inspiratory and expiratory muscle training progressively increased resistance from 30% to 75% of each patient’s maximum respiratory pressure; CG: fixed resistance of 9 cmH2O. 5 sets of 5 reps × 6 days per week × 4 months	Exp group had no significant improvement in swallowing the QOL questionnaire compared to CG.	100%
Quinn et al. 2014	7	N = 30 Age = 57 ± 10.1 Disease stage = middle stage	UHDRS-ms; UHDRS cognitive; BBS, 10 MWT (SS and FS), chair sit-to-stand test; HDQOL, EQ5D Health Index	Exp: home-based task-specific intervention program was individualized to participants’ specific activity limitations in walking, sit-to-stand transfers, and standing ability and modified to their home environments. CG: usual care. 60 min × 2 times a week × 8 weeks	Exp group showed a small effect size in all measures.	96.9%
Busse et al. 2013	7	N = 31 Age = 53.3 ± 12.5 Disease stage = early to middle	UHDRS-ms, UHDRS cognitive 10 MWT (SS and FS), Chair sit-to-stand test, Romberg test, average daily step, SF-36.	Exp: Supervised gym sessions of stationary cycling and resistance exercises and unsupervised home-based walking program consisting of (1) aerobic training at 55%–75% age-predicted maximal HR & moderate to hard levels of exertion on Borg RPE (4–6); (2) strength training for trunk/LE muscles progressed to 2 sets of 8–12 reps at 60–70% of participant’s 1 repetition max and (3) walking at moderate to somewhat hard (3–4 Borg scale) intensities; CG: usual care. 30 min × 1 time per week (gym); 2 times per week (home) × 12 Weeks.	Exp group had a significant improvement in SF-36 Mental Component Summary score and non-significant improvements in UHDRS cognitive scores and Chair sit-to-stand test.	82%
Cruickshank et al. 2018	7	N = 18 Age = 52.5 ± 5.4 Disease stage = middle stage	10-MWT (SS and FS), BBS, Chair sit-to-stand test.	Exp: supervised exercise aerobic (cycle ergometer) and resistance (machines) strengthening exercises, walking, balance, and fine motor exercises. Cognitive therapy (paper and pencil and cognitive exercises) and ADL. Gym: 60 m. once/week followed by home: 60 m. session per 3 times/week for 36 weeks. CG: Usual care medication	Exp group had no significant improvement in gait speed, BBS, and sit-to-stand test.	56%
Thompson et al., 2012	6	N = 20 Age = 53.05 ± 2.75 Disease stage = Early to middle stage	UHDRS-ms, ABC, SDMT, HVLT-R, SF-36, and HDQOL	Exp: the gym exercise comprised of supervised group sessions 5 min. warm-up, 10 min. aerobic exercise, 40 min. resistance exercise, 5 min. cool-down, once/week for 36 weeks; A tailored, self-monitored home-based exercise 3 times /week for 24 weeks and OT 1 h CG: Usual care	Exp group has significant improvement in motor function, balance, depression, cognition, and QoL	56%

Abbreviations: N: numbers; Exp: experimental group; CG: control group; HD: Huntington’s Disease; NR: not reported; UHDR-ms: Unified Huntington’s Disease Rating Scale-motor score; BBS: Berg Balance scale; ABC: Activities-Specific Balance Confidence scale; 10 MWT (SS and FS): 10 m walking test (self-selected and fast selected); WHOQOL-Bref: World Health Organization Quality of Life—Bref; HDQOL: Huntington’s Disease Health-related quality of life; SDMT: Symbol Digit Modalities Test; HVLT-R: Hopkins Verbal Learning Test-Revised.

## Data Availability

Not applicable.
